# Diagnostic value of neurofilaments in differentiating motor neuron disease from multifocal motor neuropathy

**DOI:** 10.1007/s00415-024-12355-8

**Published:** 2024-04-29

**Authors:** Camilla Wohnrade, Tabea Seeliger, Stefan Gingele, Bogdan Bjelica, Thomas Skripuletz, Susanne Petri

**Affiliations:** 1 Department of Neurology, Hannover Medical School, 30625 Hannover, Germany; 2 Center for Systems Neuroscience (ZSN) Hannover, 30559 Hannover, Germany

**Keywords:** Neurofilaments, Motor neuron disease, Amyotrophic lateral sclerosis, Multifocal motor neuropathy, Biomarker, Diagnostic performance

## Abstract

**Objective:**

To evaluate the performance of serum neurofilament light chain (NfL) and cerebrospinal fluid (CSF) phosphorylated neurofilament heavy chain (pNfH) as diagnostic biomarkers for the differentiation between motor neuron disease (MND) and multifocal motor neuropathy (MMN).

**Methods:**

This retrospective, monocentric study included 16 patients with MMN and 34 incident patients with MND. A subgroup of lower motor neuron (MN) dominant MND patients (*n* = 24) was analyzed separately. Serum NfL was measured using Ella automated immunoassay, and CSF pNfH was measured using enzyme-linked immunosorbent assay. Area under the curve (AUC), optimal cutoff values (Youden’s index), and correlations with demographic characteristics were calculated.

**Results:**

Neurofilament concentrations were significantly higher in MND compared to MMN (*p* < 0.001), and serum NfL and CSF pNfH correlated strongly with each other (Spearman’s rho 0.68, *p* < 0.001). Serum NfL (AUC 0.946, sensitivity and specificity 94%) and CSF pNfH (AUC 0.937, sensitivity 90.0%, specificity 100%) performed excellent in differentiating MND from MMN. Optimal cutoff values were ≥ 44.15 pg/mL (serum NfL) and ≥ 715.5 pg/mL (CSF pNfH), respectively. Similar results were found when restricting the MND cohort to lower MN dominant patients. Only one MMN patient had serum NfL above the cutoff. Two MND patients presented with neurofilament concentrations below the cutoffs, both featuring a slowly progressive disease.

**Conclusion:**

Neurofilaments are valuable supportive biomarkers for the differentiation between MND and MMN. Serum NfL and CSF pNfH perform similarly well and elevated neurofilaments in case of diagnostic uncertainty underpin MND diagnosis.

## Introduction

Amyotrophic lateral sclerosis (ALS) is a fatal neurodegenerative disorder characterized by motor neuron (MN) loss in the primary motor cortex, brainstem, and spinal cord. Patients typically suffer from progressive weakness of voluntary muscles due to lower motor neuron degeneration and increased muscle tone due to upper motor neuron loss [1, 2]. Because of its heterogeneous phenotypic presentation, in particular regarding disease phenotypes without clinically apparent upper motor neuron signs, the diagnosis oftentimes can be challenging leading to a diagnostic delay of 10 to 16 months [3, 4]. Until recently the diagnosis of ALS according to the revised El Escorial criteria (2000) and Awaji criteria (2008) relied on clinical signs of upper and lower motor neuron dysfunction possibly supported by electrophysiological signs of lower motor neuron dysfunction and the exclusion of mimicking diseases [4, 5]. With the introduction of the Gold Coast criteria in 2019, the presence of progressive motor impairment with lower motor neuron dysfunction in at least two body regions is sufficient to diagnose ALS [6]. This increased the diagnostic sensitivity especially in lower MN dominant ALS and the ALS variant progressive muscular atrophy (PMA) with pure lower motor neuron affection [7]. Nevertheless, the distinction between ALS and common mimicking diseases such as inflammatory polyneuropathies remains complex. Of the inflammatory polyneuropathies, multifocal motor neuropathy (MMN) presents with pure, progressive, focal motor impairment and, therefore, constitutes a plausible differential diagnosis to ALS/MND. MMN is a rare disorder with a prevalence of < 2 per 100 000. As pathophysiological correlate of peripheral nerve demyelination, an antibody-mediated attack of the nodes of Ranvier and/or the paranodal region (nodopathy/paranodopathy) is presumed [8, 9]. Accordingly, serum IgM antibodies to ganglioside GM1 (anti-GM1 antibodies) are present in about half of the cases of MMN, but they are not specific and occur in healthy controls as well as in ALS [10, 11]. The characteristic electrophysiological finding of MMN is motor conduction blocks outside of typical entrapment sites. However, conduction blocks can be absent or elude routine nerve conduction studies due to proximal location [9, 12]. First-line treatment of MMN is repeated infusions of intravenous immunoglobulins (IVIg) [13, 14], and in case of insufficient treatment response, other immunomodulatory substances are used. Especially in the absence of anti-GM1 antibodies and inconclusive electrophysiological studies, MMN can be confounded with a lower MN dominant ALS or PMA with pivotal implications regarding prognosis and treatment options.

The development of (fluid) biomarkers to increase diagnostic accuracy has been an essential objective in ALS research in the recent past and the Airlie House guidelines recommended that biomarkers should be included as a best practice for clinical trial design [15]. As one of the most promising biomarkers, neurofilaments (Nf) and their performance in distinguishing ALS from ALS mimics have been studied extensively. Nf are cylindrical cytoskeletal proteins that consist of four subunits: neurofilament light chain, middle chain, heavy chain and α-internexin [16, 17]. They are expressed solely in neurons and are considered as markers of axonal damage, as their expression in axons is particularly high. Neurofilament light chain (NfL) is the most abundant and most soluble subunit with stable concentrations in biofluids making measurements more reliable compared to the other subunits. Phosphorylated neurofilament heavy chain (pNfH), on the other hand, contains abundant phosphorylation sites, which are important for structural stability and protect the protein from degradation. Small amounts of Nf are constantly released into cerebrospinal fluid (CSF) and blood in an age-dependent manner, but concentrations increase to a various extent in different neurological conditions including traumatic, inflammatory, and degenerative disorders [18, 19]. Especially in rapidly progressive diseases such as HIV-associated dementia, Creutzfeldt–Jakob disease or amyotrophic lateral sclerosis CSF Nf concentrations are high [18]. While their diagnostic and prognostic value in ALS nowadays is undisputable [20], different studies have reported a diagnostic sensitivity ranging from 76–100% and a specificity of 75–92% [21–24] depending on the composition of the respective control cohort. To detail the diagnostic value of neurofilaments, it is necessary to define the target population carefully and focus on conditions with similar clinical characteristics to MND. Therefore, the aim of this study was to evaluate the performance of serum NfL and CSF pNfH as diagnostic biomarkers for the differentiation between MND and MMN.

## Methods

### Study design and participants

This retrospective, monocentric study included patients with the diagnosis of MMN or MND, who underwent Nf sampling in CSF and serum at the Department of Neurology at Hannover Medical School (Hannover, Germany) between 2008 and 2022. Participants aged 18 years and over presented consecutively in an in-/outpatient setting either for primary diagnostic evaluation of suspected motor neuropathy or to confirm or reevaluate a presumed MND or MMN diagnosis. MMN was diagnosed based on the European Federation of Neurological Societies/Peripheral Nerve Society (EFNS/PNS) criteria from 2010 [14]. Briefly, presence of a slowly progressive, focal, asymmetric limb weakness without sensory abnormalities was mandatory, and responsiveness to immunomodulatory treatment was evaluated as supportive. From the total cohort of 48 cases with an initial MMN diagnosis in *n* = 9 cases, CSF sampling was not performed in our clinic, leaving *n* = 39 potentially eligible cases. A subset of MMN patients had received immunomodulatory treatment previous to sample collection and no CSF sampling was performed (*n* = 6). The diagnosis of MND was prompted by an experienced MND specialist neurologist according to the revised El Escorial criteria, the PLS (primary lateral sclerosis) 2020 consensus diagnostic criteria [25], and the Gold Coast criteria (in cases with PMA) [6]. As numbers of MND patients exceeded MMN patients, we performed backwards selection of MND patients starting in October 2022, which was concluded, when the year 2020 and a similar number of potentially eligible MND patients compared to MMN patients was reached (*n* = 38). In detail, from a total cohort of *n* = 169 cases with MND diagnosis (October 2022 until January 2020), in *n* = 38 cases, neurofilament sampling was performed (potentially eligible cases). A subset of MND patients had received immunomodulatory treatment due to suspected inflammatory polyneuropathy (*n* = 5), but CSF and serum sampling had been performed before treatment initiation in these cases. This study report was structured following the “Standards for Reporting Diagnostic accuracy studies” (STARD) updated in 2015 [26].

### Clinical parameters

Participant and disease characteristics (sex, body mass index (BMI), age at sampling, time of disease onset, site of disease onset, Medical Research Council (MRC) sum score, ALS Functional Rating Scale revised (ALSFRSr), electrophysiological testing, anti-GM1 antibody testing, final diagnosis, immunomodulatory treatment, clinical follow up) were retrieved from medical records. Functional evaluation (MRC sum score [27], ALSFRSr [28]) was performed during the same inpatient visit as sample collection or within 12 weeks thereafter. The rate of change in ALSFRSr (ALSFRSr slope) was used to determine the rate of disease progression at baseline for MND patients. This was calculated as decline in 48-ALSFRSr score divided by the number of months between symptom onset and ALSFRSr assessment (with an interval of at least three months) [29]. To compare severity between the two diseases, an MRC sum score was calculated by assessing eight muscle groups bilaterally. The muscle groups comprised shoulder abductors, elbow flexors, wrist extensors, thumb abductors, hip flexors, knee extensors, foot and big toe dorsiflexors, resulting in a maximum score of 80 points (0–5 points per muscle group and side).

To further narrow down the study population to patients with a likely MMN differential diagnosis, a subset of lower MN dominant MND patients was defined: for attribution to the lower MN dominant MND subgroup, electrophysiological signs of active lower MN denervation needed to be present in two or more regions in the absence of prolonged central motor conduction time, and clinical pyramidal tract signs (spastic increase in muscle tone, clonic deep tendon reflexes, extensor plantar response, Hoffmann reflex). Preserved deep tendon reflexes in a paretic limb were present in all but one MND patients and did not constitute an exclusion criterion for the lower MN dominant MND subgroup.

### Sample collection Nf quantification

Paired CSF and serum samples were obtained by lumbar puncture and consecutive venipuncture performed during routine clinical workup at the Department of Neurology, Hannover Medical School, Hannover, Germany between 2008 and 2022. Blood and CSF samples were collected in serum separator tubes and polypropylene centrifuge tubes, respectively. Serum was centrifuged at 3500 rpm for 10 min, CSF was centrifuged at 900 rpm for 15 min, both at 4 °C, and the supernatant was aliquoted into sterile microtubes for storage at -80 °C within 2 h. Samples were thawed, aliquoted into polypropylene tubes, refrozen and shipped on dry ice to Neurochemistry Laboratory at Ulm University, Ulm, Germany in 2022 and 2023, where NfL and pNfH analyses were performed.

NfL in serum was measured using Ella automated immunoassay system (bio-techne GmbH, Minneapolis, USA), while pNfH in CSF was measured using enzyme-linked immunosorbent assay (Sandwich ELISA, BioVendor R&D, Karasek, Czech Republic) according to the manufacturer’s instructions. The range of the Ella automated immunoassay was 2.7–10.29 pg/mL with a sensitivity of 1.1 pg/mL. For pNfH, the calibration range was 62.5–4000 pg/mL and the CSF was diluted threefold. Six MMN patients and one MND patient had CSF pNfH concentrations below the detection limit of the assay. These samples were assigned the concentration of the lower calibration curve limit (188 pg/mL). Regarding serum NfL, one MMN patient had concentrations below the detection limit of the assay, here, the extrapolated value (simple plex runner software) was used for analyses.

### Statistical analyses

Statistical analyses were performed using IBM SPSS Statistics version 29 (IBM, Armonk, NY, USA). Normal distribution was assessed visually by quantile–quantile plot analysis and by Kolmogorov–Smirnov test. Apart from age at sample collection, data were not normally distributed. Heteroscedasticity was tested using Levene’s test, which revealed that variances of Nf concentrations were not homogenous between MMN and MND patients. Accordingly, non-parametric tests were chosen for analyses including Nf concentrations. Regarding age, BMI, and MRC sum score, variances between MMN and MND patients were homogenous, so that parametric tests were applied. Descriptive statistics were calculated and depicted as number, percentage, median and range or mean and standard deviation. Mann–Whitney *U* test and *t* test for independent sampling were used to determine differences in metric variables between two groups, as appropriate. Chi-squared and Fisher’s exact tests were used to determine associations between categorical variables. Bivariate correlations were studied by means of Pearson or Spearman rank correlation coefficient. Receiver operating characteristic (ROC) curves were generated to evaluate the performance of NfL and pNfH to distinguish between MMN and MND. The area under the (ROC) curve (AUC) was composed and an AUC of > 0.9 was considered as excellent, an AUC of > 0.8 was considered as good performance. Youden’s index highest value and lowest value of closest-to-top-left analysis were used to determine the optimal cutoff for serum NfL and CSF pNfH as well as sensitivity and specificity. For all analyses, significance levels were set at *p* < 0.05 (two-tailed).

## Results

### Participant characteristics

**Fig. 1:**
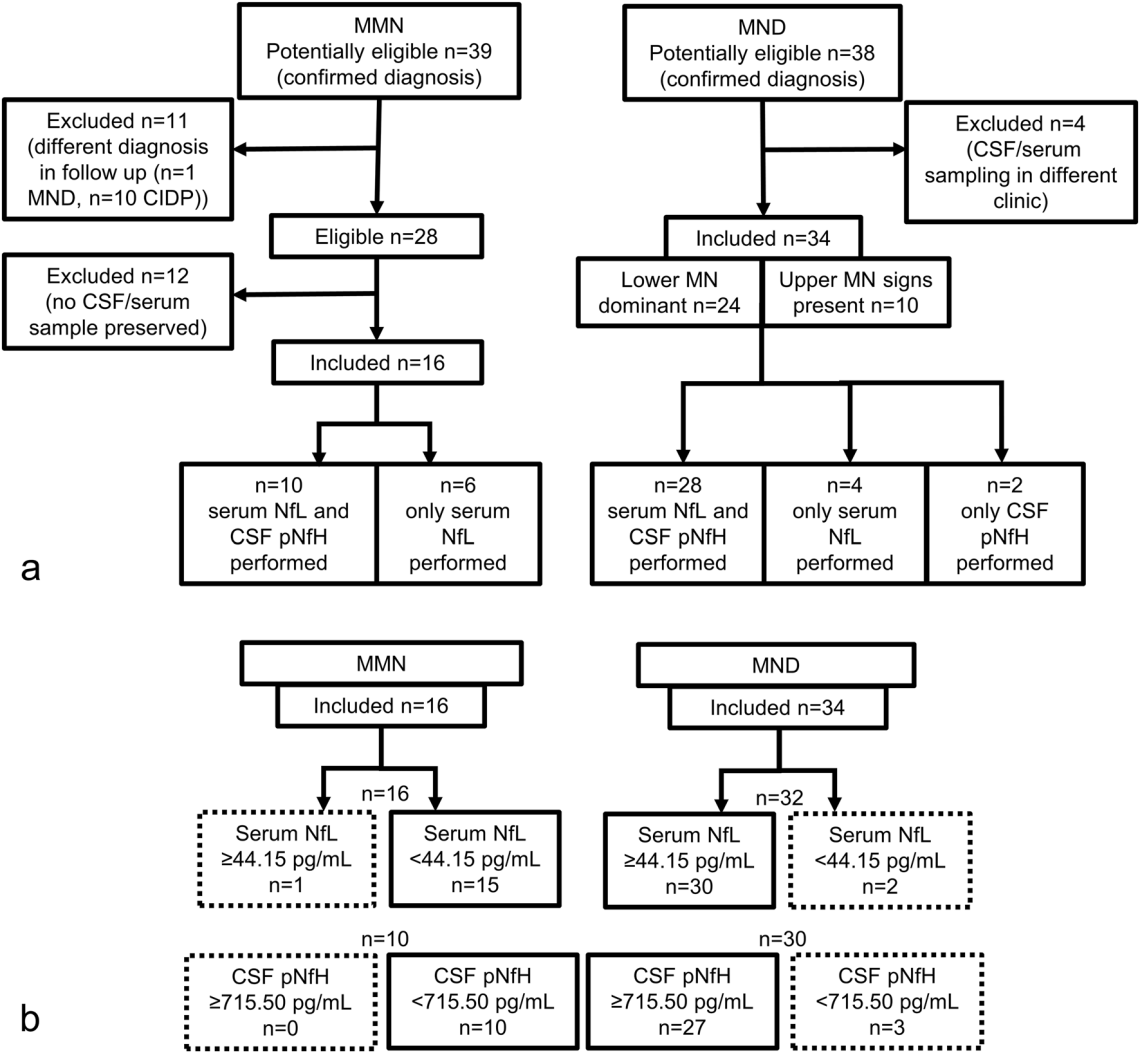
Participant flow. **a** Flow of participants from screening until inclusion and number of serum and CSF samples analyzed. **b** Cross-tabulation of the clinical diagnosis by Nf concentrations. Dotted boxes constitute false positives and false negatives, whereas boxes with solid border represent true positives and true negatives applying the estimated cutoffs. *MMN* multifocal motor neuropathy, *MND* motor neuron disease, *CIDP* chronic inflammatory demyelinating polyneuropathy, *CSF* cerebrospinal fluid, *MN* motor neuron, *NfL* neurofilament light chain, *pNfH* phosphorylated neurofilament heavy chain

A total of 39 MMN patients and 38 MND patients were identified as potentially eligible. Out of 28 eligible MMN patients, *n* = 16 serum and *n* = 10 CSF samples had been preserved and were analyzed. Thirty-four MND patients were confirmed eligible and included in the analyses (for the detailed participant flow see Fig. 1).

Table 1 shows the characteristics of MND and MMN participants. The MND cohort included one patient diagnosed with PMA, five patients diagnosed with PLS, and one patient with ALS-FTD (frontotemporal dementia). Genetic testing was performed in 12 MND patients, a *C9orf72* hexanucleotide repeat expansion was found in two, mutations in *SOD1, FUS* and *FIG4* in one patient each. Of the MMN patients, eleven (68.8%) had characteristic conduction blocks in the electrophysiological evaluation, five (31.3%) were anti-GM1 antibody positive. There were no significant differences regarding gender or BMI between all MND patients, lower MN dominant MND patients and MMN patients. MND patients were significantly older at CSF/serum sampling compared to MMN patients, while MMN patients had a significantly longer disease duration. MRC sum score as exploratory disease-spanning measure of motor impairment did not differ between the groups. MND patients (whole cohort as well as lower MN dominant subgroup) had a median ALSFRSr of 40 and did not differ with regard to age and disease duration. However, ALSFRSr slope was steeper in lower MN dominant MND patients and spinal onset was also more frequent in this subgroup. All MND patients who had received immunomodulatory therapy on the suspicion of an inflammatory motor neuropathy were assigned to the lower MN dominant subgroup. Four had received three to five cycles of IVIg, and one had received intravenous methylprednisolone. Of the pretreated MMN patients, all six had received IVIg over a period of 1 year to 16 years, one patient had additionally been treated with mycophenolate mofetil and one patient with azathioprine, each over a period of one year at blood sampling.

**Table 1:** Participant characteristics

	MND, all n = 34		Lower MN dominant MND n = 24		MMN n = 16		*p*	*p*	*p*
							All MND versus MMN	Lower MN MND versus MMN	Lower MN MND versus remaining MND
Female N (%)	14 (41.2)	*n* = 34	11 (45.8)	*n* = 24	8 (50.0)	*n* = 16	0.697	0.796	0.529
BMI (kg/cm^2^), mean (std)	26.4 (5.6)	*n* = 34	26.19 (5.37)	*n* = 24	29.5 (5.5)	*n* = 16	0.070	0.070	1.000
Age at sampling (years), median (min–max)	62 (40–77)	*n* = 34	63 (40–77)	*n* = 24	55 (31–77)	*n* = 16			
Age at sampling (years), mean (std)	61.47 (8.65)		62.04 (9.50)		53.00 (12.45)		**0.007**	**0.013**	0.559
Disease duration at sampling (months), median (min–max)	9 (2–72)	*n* = 33	9 (2–27)	*n* = 23	29.5 (6–216)	*n* = 16	**0.005**	**0.002**	0.105
MRC sum score at sampling, median (min–max)	77 (12–80)	*n* = 28	77 (12–80)	*n* = 21	77 (64–80)	*n* = 16			
MRC sum score at sampling, mean (std)	72.00 (14.57)		71.48 (15.44)		74.25 (5.69)		0.558	0.500	0.749
ALSFRSr at sampling, median (min–max)	40 (20–47)	*n* = 32	39.5 (20–47)	*n* = 22					0.704
ALSFRSr slope (points/month), median (min–max)	0.51 (0.13–5.33)	*n* = 32	0.67 (0.13–3.00)	*n* = 22					**0.025**
Site of onset, spinal N (%)	24 (70.6)		20 (83.3)						**0.019**
Immune therapy before sampling N (%)	5 (14.7)		5 (20.8)		6 (37.5)				0.535
Serum NfL (pg/mL), median (min–max)	81.00 (22.00–605.00)	*n* = 31	78.50 (22.00–382.00)	*n* = 22	15.95 (4.25–118.00)	*n* = 16	** < 0.001**	** < 0.001**	0.334
Serum NfL (pg/mL), mean (std)	122.16 (119.08)		104.00 (84.09)		22.62 (26.73)				
CSF pNfH (pg/mL), median (min–max)	1867.50 (169.00–7653.00)	*n* = 30	1872.00 (169.00–7653.00)	*n* = 21	188.00 (< 188.00–564.00)	*n* = 10	** < 0.001**	** < 0.001**	0.722
CSF pNfH (pg/mL), mean (std)	2429.50 (2046.84)		2505.43 (2100.57)		283.50 (150.41)				

Significant *p* values are printed in bold type

*MND* motor neuron disease, *MN* motor neuron, *MMN* multifocal motor neuropathy, *BMI* body mass index, *std* standard deviation, *min* minimum, *max* maximum, *MRC* Medical Research Council, *ALSFRSr* ALS Functional Rating Scale revised, *NfL* neurofilament light chain, *CSF* cerebrospinal fluid, *pNfH* phosphorylated neurofilament heavy chain

### Nf concentrations and correlations

**Fig. 2:**
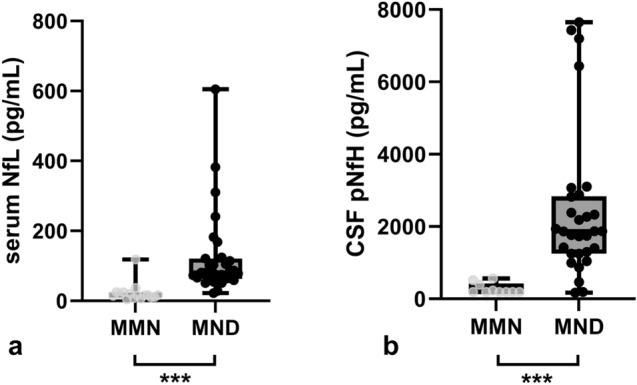
Neurofilament concentrations in MMN versus MND. Depicted are median and range for serum NfL and CSF pNfH concentrations. Each grey/black dot represents an individual MMN/MND patient. MND patients had significantly higher Nf concentrations compared to MMN patients (*p* < 0.001). **a** Serum NfL in MMN versus MND. **b** CSF pNfH in MMN versus MND. *MMN* multifocal motor neuropathy, *MND* motor neuron disease, *NfL* neurofilament light chain, *CSF* cerebrospinal fluid, *pNfH* phosphorylated neurofilament heavy chain, ***p < 0.001

Serum NfL and CSF pNfH concentrations were significantly higher in MND patients compared to MMN patients (*p* < 0.001) (Fig. 2). The same applied if lower MN dominant MND patients were compared to MMN patients (*p* < 0.001). Between lower MN dominant MND patients and the remaining MND patients, there were no significant differences in serum NfL and CSF pNfH concentrations.

Serum NfL correlated strongly with CSF pNfH in the whole cohort (MND and MMN patients combined, Spearman’s rho 0.796, *p* < 0.001, *n* = 37). In the MND cohort, Nf in serum and CSF correlated more strongly in the cohort of MND cases with lower and upper MN signs (Spearman’s rho 0.714, *p* = 0.047, *n* = 8) compared to the lower motor neuron dominant cases (Spearman’s rho 0.643, *p* = 0.03, *n* = 19). Both, serum NfL and CSF pNfH correlated with age in the MMN but not the MND cohort. In the MND cohort, no correlation was found between Nf and disease duration or ALSFRSr. However, there was a near significant association observed between Nf and ALSFRSr slope and MRC sum score. In the MMN cohort, CSF pNfH showed a significant positive correlation with MRC sum score (Table 2).

**Table 2:** Correlations between neurofilament concentrations and participant characteristics

MND	Serum NfL			CSF pNfH		
	Coefficient	*p*	*n*	Coefficient	*p*	*n*
Age at sampling	0.093	0.681	31	(− 0.100)	0.600	30
BMI at sampling	0.317	0.083	31	0.297	0.112	30
disease duration	(− 0.189)	0.317	30	(− 0.117)	0.545	29
MRC sum score	(− 0.341)	0.095	25	(− 0.371)	0.068	25
ALSFRSr	(− 0.137)	0.477	29	(− 0.102)	0.605	28
ALSFRSr slope	0.203	0.290	29	0.332	0.084	28
Serum NfL	1.000		31	**0.626**	** < 0.001**	**27**
CSF pNfH	**0.626**	** < 0.001**	**27**	1.000		30

MMN	Serum NfL			CSF pNfH		
	Coefficient	*p*	*n*	Coefficient	*p*	*n*
Age at sampling	**0.717**	**0.002**	**16**	**0.632**	**0.050**	**10**
BMI at sampling	0.203	0.450	16	0.232	0.519	10
disease duration	(− 0.332)	0.209	16	(− 0.270)	0.450	10
MRC sum score	0.236	0.379	16	**0.770**	**0.009**	**10**
Serum NfL	1.000		16	**0.683**	**0.030**	**10**
CSF pNfH	**0.683**	**0.030**	**16**	1.000		10

Significant correlations are printed in bold type

*MND* motor neuron disease, *BMI* body mass index, *MRC* Medical Research Council, *ALS* amyotrophic lateral sclerosis, *ALSFRSr* ALS Functional Rating Scale revised, *NfL* neurofilament light chain, *CSF* cerebrospinal fluid, *pNfH* phosphorylated neurofilament heavy chain, *MMN* multifocal motor neuropathy

### Diagnostic performance of Nf

#### Serum NfL in MMN versus MND

**Fig. 3:**
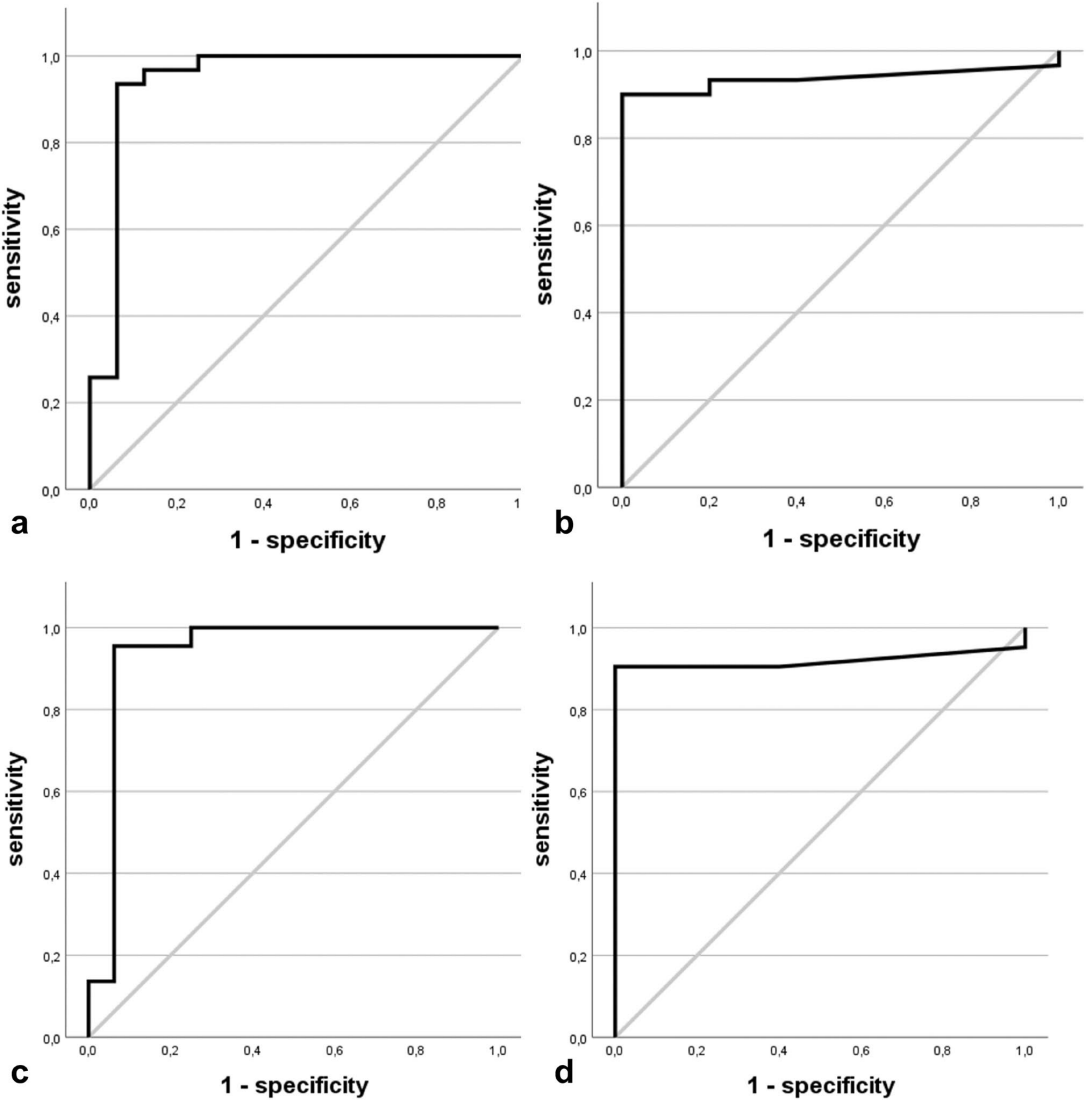
ROC curves for the evaluation of diagnostic accuracy of serum NfL and CSF pNfH to discriminate MND from MMN **a** ROC curve of serum NfL in all MND patients (*n* = 32) versus MMN patients (*n* = 16). **b** ROC curve of CSF pNfH in all MND patients (*n* = 30) versus MMN patients (*n* = 10). **c** ROC curve of serum NfL in lower MN dominant MND patients (*n* = 23) versus MMN patients (*n* = 16). **d** ROC curve of CSF pNfH in lower MN dominant MND patients (*n* = 21) versus MMN patients (*n* = 10). *MND* motor neuron disease, *MMN* multifocal motor neuropathy, *NfL* neurofilament light chain, *CSF* cerebrospinal fluid, *pNfH* phosphorylated neurofilament heavy chain

The ROC curve for serum NfL had an AUC of 0.946, confirming an excellent ability of serum NfL to differentiate between MND and MMN (*p* < 0.001; 95% confidence interval (CI) 0.856–1.035). A cutoff of ≥ 44.15 pg/mL gave a sensitivity of 93.5% and a specificity of 93.7% for identifying patients with MND (Fig. 3a). After limiting the MND cohort to lower MN dominant MND patients, AUC decreased to 0.938 (*p* < 0.001, CI 0.833—1.042). The same cutoff of ≥ 44.15 pg/mL resulted in a sensitivity of 95.5% and a specificity of 93.7% for identifying patients with MND (Fig. 3c).

#### CSF pNfH in MMN versus MND

The ROC curve for CSF pNfH showed an excellent ability to differentiate between MND and MMN (AUC 0.937, CI 0.856–1.017, *p* < 0.001). The optimal cutoff was estimated as ≥ 715.50 pg/mL, which gave a sensitivity of 90.0% and 100% specificity for identifying patients with MND (Fig. 3b). In the lower MN dominant MND cohort, CSF pNfH had a similar AUC of 0.919 (*p* < 0.001, CI 0.808–1.030) corresponding to a very good discriminatory value. The same cutoff of ≥ 715.50 pg/mL yielded a sensitivity of 90.5% and a specificity of 100% (Fig. 3d).

### Characterization of MND patients below and MMN patients above the estimated cutoffs

Appling the estimated cutoff, there was only one patient diagnosed with MMN with a serum NfL concentration above 44.15 pg/mL (118 pg/mL). This patient was anti-GM1 antibody positive (low serum titer) and had conduction blocks (left median and ulnar nerve) supporting the diagnosis of MMN. The patient was male, aged 57 years at the time of sampling and presented with minor distal pareses of the upper extremities (MRC sum score 78 points). Regarding comorbidities, he exhibited a BMI of 34.3 and had been diagnosed with diabetes mellitus type 2. Nerve conduction studies detected slight sensory involvement in the lower extremity nerves possibly indicative for diabetic polyneuropathy. During treatment with IVIg, the patient experienced a subjective improvement of muscle strength and MRC sum score remained stable (77 points after 16 months of treatment). CSF pNfH in this patient was measurable, but below the estimated cutoff of ≥ 715.50 pg/mL. None of the ten MMN patients with preserved CSF samples had pNfH values above the estimated cutoff.

Two MND patients presented with serum NfL values below the estimated cutoff. They were 68 years (male) and 57 years (female) old at sampling and both had a slowly progressive MN disease: the male patient reported a stable disease over a period of three years with an ALSFRSr remaining above 40 points and an isolated atrophic paresis of the left upper extremity. He received no immunomodulatory treatment, but used commercially available tauroursodeoxycholic acid as supplement and participated in an interventional clinical trial. Anti-GM1 antibodies and conduction blocks were absent in this patient, but he presented with ubiquitously increased deep tendon reflexes. The female patient was diagnosed with the clinical MND variant of PLS, had a disease duration of 23 months at sampling and an ALSFRSr slope of 0.21 points/month. The same two MND patients also had CSF pNfH values below the estimated cutoff. A third MND patient presented with CSF pNfH < 715.50 pg/mL. This patient was male, aged 69 years at sampling, and presented with the clinical MND variant of PMA. Disease duration at sampling was four months and ALSFRSr slope was 1.18 points/month. Serum NfL was not measured due to technical issues. However, in a subsequent analysis, seven months later in a different laboratory, serum NfL concentration was estimated 307 pg/mL, suggesting an analytical error during the first analysis of CSF pNfH.

## Discussion

In this retrospective diagnostic accuracy study including 50 MMN and MND patients, serum NfL and CSF pNfH performed excellent in differentiating MND from one of its most challenging mimicking diseases, MMN.

Diagnostic performance of neurofilaments was better than previously reported in ALS for serum/plasma NfL [21–23, 30, 31] and CSF pNfH [30–33]. The refinement of the target population by limiting MND mimics to MMN patients and excluding for example demyelinating polyneuropathies may have improved diagnostic performance. However, Kläppe et al. reported a similar AUC for CSF pNfH and a slightly better AUC for serum NfL in differentiating ALS from ALS mimics including among others (motor) neuropathies, myopathies, spinal stenosis, and neuroborreliosis [34]. So far, only one study evaluated diagnostic performance of plasma and CSF NfL in MMN versus MND and reported AUCs of 0.9 and 0.94, respectively. The study included only *n* = 8 MMN patients [23]. In our study, diagnostic performance of serum NfL and CSF pNfH was equivalent, which is in line with the literature [30, 34–36]. This was further supported by a strong positive correlation of serum NfL and CSF pNfH (Spearman’s rho 0.796).

In our study, discriminative ability of serum NfL and CSF pNfH decreased only slightly, when a subgroup of lower MN dominant MND patients was defined and compared to MMN patients. Neurofilament concentrations did not differ significantly between the two MND groups, while ALSFRSr slope was higher (median 0.67 versus 0.30 points/month) in the lower MN dominant subgroup. Previous studies found a positive correlation of CSF NfL concentrations with the extent of lower MN involvement measured by electromyography [24], and there is vast evidence for their unfavorable association with disease progression and survival [17, 20, 24, 37–39]. It is, therefore, surprising that Nf concentrations were not increased in this subgroup. However, there were less patients with bulbar onset (4 out of 24 versus 6 out of 10) in the lower MN dominant subgroup, which may have counterbalanced increased Nf concentrations [30, 38]. Also, the association between Nf concentrations and involvement of upper versus lower motor neurons is controversial [17].

The estimated optimal serum NfL cutoff (≥ 44.15 pg/mL) for diagnosing MND was lower than reported earlier [36, 40] or compared to studies including various mimicking diseases [21, 22, 30]. More recent studies (Kläppe et al.: cutoff 56.4 pg/mL, Verde et al.: cutoff 49–62 pg/mL [34, 41]) as well as studies focusing on selected, well-characterized mimics (Gille et al.: 55 pg/mL versus hereditary spastic paraplegia [40]) found similar cutoffs. For CSF pNfH, our optimal cutoff of ≥ 715.50 pg/mL was well in the range of previously reported cutoffs (Kläppe et al.: 726 pg/mL; Poesen et al.: 768 pg/mL; Steinacker et al.: 560 pg/mL; Chen et al.: 437 ng/L; Li et al.: 395 pg/mL, Li et al.: 1104 pg/mL [32–34, 42–44]). Of note, the one MMN patient with serum NfL above the optimal cutoff presented with comorbid diabetes mellitus type 2. In a large population-based study conducted in the United States, patients with diabetes mellitus exhibited higher serum NfL concentrations compared to non-diabetic participants [45], resembling the Swiss-atrial fibrillation study [46]. Further, NfL concentrations are increased in patients who develop diabetic neuropathy [47]. Thus, high serum NfL concentrations may have been the result of comorbid diabetes mellitus (and possibly diabetic neuropathy) in this patient, and diabetes should be taken into account as confounding variable when applying neurofilaments as diagnostic biomarkers. Both MND patients with serum NfL and CSF pNfH values below the cutoffs featured a slowly progressive disease with an ALSFRSr slope < 0.25 points/month. As serum NfL and CSF pNfH are strongly associated with disease progression [18, 30, 34, 36–38, 48], relatively benign disease courses as the ones described above may not match the estimated cutoffs and be rated as false negatives.

Surprisingly, we did not find a correlation of Nf concentrations with age or ALSFRSr slope in our cohort though significance was slightly missed for ALSFRSr slope and CSF pNfH. Even though Nf correlate with age in healthy controls and other neurological diseases [49, 50], the evidence for MND patients is inconclusive [38, 51, 52] and it has been postulated that the massive elevation of Nf in MND extinguishes the mild elevation due to age in MND [17]. Accordingly, in the MMN group, there was a significant positive correlation between age and serum NfL/CSF pNfH. For the same reason, the imbalance in age between MND and MMN patients in our cohort should not have affected our analyses.

Most studies found no correlation between Nf concentrations and functional measures such as ALSFRSr and MRC scores [31, 41, 48, 53–55], which is in line with our findings in our MND cohort. Regarding ALSFRSr slope, significances might have been missed in our study due to the low number of included patients. But there was a trend toward a positive correlation between ALSFRSr slope and CSF pNfH, and patients with particularly high or low Nf concentrations (as described for the two MND patients below the cutoff) presented with accordingly fast or slow disease progression.

Regarding the sensitivity of the ELISA assays for detection of neurofilaments, the high sensitivity Ella automated immunoassay used for detection of serum NfL exhibits a detection limit of 1.1 pg/mL, which exceeds earlier ELISA assays [56]. However, sensitivity falls short of the widely used SIMOA assay with a sensitivity below pg/mL concentrations [57]. For samples that are expected to have high NfL concentrations, as it is the case in motor neuron disease, ELISA assays seem to be sufficient. Accordingly, in all serum samples, even the samples derived from MMN patients with lower neurofilament concentrations, NfL concentrations were above the detection limit. CSF neurofilament concentrations are about 40-fold higher compared to serum concentrations [18], making ELISA assays sufficiently sensitive methods for their quantification.

The main strength of this study is the refinement and careful definition of the target population by focusing on MMN as one of the most challenging mimics of lower MN dominant MND. To date, to the best of our knowledge, only two studies evaluated diagnostic performance of Nf in MMN including three [58] and eight [23] patients, respectively. Only the latter compared Nf concentrations to MND patients. Here, we were able to analyze CSF/serum Nf concentrations of a comparably large cohort of 16 MMN patients. The limitations of this study include its retrospective and monocentric design and the low number of participants, due to which further subgroup analyses were not feasible. Further, some of the CSF/serum samples had been stored for several years (maximum 14 years) and underwent one or two freeze–thaw cycles before Nf measurement. However, previous studies found no alteration of CSF Nf concentrations depending on freezer storage time [30] or the number of freeze–thaw cycles [59]. To compare overall disease severity between diseases, we calculated an MRC sum score, which is not validated in MND and does not comprise bulbar function or respiratory impairment. Therefore, conclusions derived from disease severity correlations are limited. Clinical characteristics differed between MMN and MND patients, in particular, MMN patients were younger and had longer disease duration compared to MND patients. As discussed above, the difference in age should not have affected our analyses. Regarding disease duration, Nf concentrations have been shown to be relatively stable throughout the course of MND [40, 41, 60, 61] suggesting that disease duration may not have a significant impact on diagnostic accuracy of Nf in MND. However, we cannot exclude that disease duration may impact Nf concentrations in MMN and limit diagnostic accuracy in early disease stages. Moreover, clinical characteristics (gender, site of onset, ALSFRSr) of our MND cohort were similar to three recently published large German ALS cohorts [37, 62, 63]. Even though MND patients in our cohort were younger—most likely due to inclusion at time of diagnosis and not later during the disease course—our results should be applicable to the German ALS/MND population. However, as source of potential bias, selection bias has to be considered, as we recruited patients exclusively at a tertiary referral hospital, which might have led to an overestimation/underestimation of disease severity and Nf concentrations. Further, it would have been desirable to evaluate a complementary role of Troponin T and Creatine Kinase MB isoenzyme for the differential diagnosis of MND and MMN, especially as recent studies suggest that these biomarkers represent lower motor neuron involvement and correlate with bulbar involvement [63, 64]. However, this was out of the scope of this study.

In conclusion, this study provides evidence that Nf are useful biomarkers to distinguish MMN from MND. While serum NfL and CSF pNfH performed equally well, caution has to be exercised with regard to comorbidities potentially influencing Nf concentration. Even though high Nf concentrations in serum and CSF should not be considered absolute, high Nf concentrations in case of diagnostic uncertainty would make MND diagnosis very likely.

## Data Availability

Deidentified data are available from the corresponding author upon reasonable request.
