# Correction to: Diagnostic value of neurofilaments in differentiating motor neuron disease from multifocal motor neuropathy

**DOI:** 10.1007/s00415-024-12491-1

**Published:** 2024-07-28

**Authors:** Camilla Wohnrade, Tabea Seeliger, Stefan Gingele, Bogdan Bjelica, Thomas Skripuletz, Susanne Petri

**Affiliations:** 1 Department of Neurology, Hannover Medical School, 30625 Hannover, Germany; 2 Center for Systems Neuroscience (ZSN) Hannover, 30559 Hannover, Germany

**Correction to: Journal of Neurology** 10.1007/s00415-024-12355-8

The originally published version of this article unfortunately contained an error regarding the assay range of the Ella automated immunoassay. The range of the assay specified in the methods section should correctly be 2.7 - 10,290 pg/mL instead of 2.7 -10.29 pg/mL, which makes a difference of three orders of magnitude.

In the section ‘’Sample collection Nf quantification’’, second sentence of the second paragraph which previously read

The range of the Ella automated immunoassay was 2.7–10.29 pg/mL with a sensitivity of 1.1 pg/mL.

Should have read

The range of the Ella automated immunoassay was 2.7–10,290 pg/mL with a sensitivity of 1.1 pg/mL.

